# Triglyceride glucose index versus triglyceride glucose-BMI index in non-alcoholic fatty liver disease: A correlation with steatosis and fibrosis

**DOI:** 10.6026/973206300222092

**Published:** 2026-04-30

**Authors:** Aditya Joshi, Deepak Tangadi, Ankit Kumar Tiwari

**Affiliations:** 1 Department of Medicine, Government Medical College, Jammu & Kashmir, India; 2 Department of Biochemistry, SSIMS & Research Centre, Davangere, Karnataka, India; 3 Department of Biochemistry, Autonomous State Medical College, Kaushambi, Uttar Pradesh, India

**Keywords:** Non-alcoholic fatty liver disease, triglyceride glucose index, BMI index, steatosis

## Abstract

Non-alcoholic fatty liver disease (NAFLD) links to insulin resistance and cardiometabolic risk, yet cost-effective markers like TyG and TyG-BMI indices for predicting steatosis and fibrosis severity remain under-evaluated in resource-limited settings. This retrospective cross-sectional study at GMC Jammu analyzed 500 NAFLD patients over 1 year, comparing TyG versus TyG-BMI diagnostic value against ultrasonographic steatosis grades and elastographic fibrosis scores using clinical/biochemical records. Both indices rose significantly with advancing steatosis and fibrosis, with TyG-BMI showing stronger correlations with hepatic severity than TyG alone, reflecting the integrated adiposity measures. TyG-BMI demonstrated enhanced predictive performance for NAFLD progression, offering a simple, non-invasive alternative to imaging in high-burden populations. This validates TyG-BMI as a superior, affordable surrogate for risk stratification and NAFLD management in resource-constrained clinical practice.

## Background:

NAFLD is one of the most prevalent chronic liver diseases in the global population and is now becoming widely acknowledged as the hepatic expression of the metabolic syndrome. It includes a continuum of easy steatosis to non-alcoholic steatohepatitis, fibrosis and cirrhosis and hepatocellular carcinoma. The increasing prevalence of obesity, diabetes and sedentary lifestyles in the world has resulted in an equivalent rise in NAFLD burden and it has become one of the primary causes of concern in the field of public health [1, 2]. According to recent epidemiological statistics, NAFLD occurs in almost a quarter of the adult world population, with even greater prevalence in South Asian populations, since they are more metabolically susceptible [3]. It is assumed that insulin resistance is the primary pathophysiological process involved in NAFLD. It further enhances lipolysis, hepatic triglyceride accumulation, oxidative stress and the release of inflammatory cytokines, ultimately leading to hepatocellular injury and fibrosis [4, 5]. Since insulin resistance is a major factor in the pathogenesis of NAFLD, surrogate measures of metabolic impairment could be valuable for early diagnosis and prognosis. The triglyceride- glucose (TyG) index, calculated as the ratio of fasting triglyceride and glucose levels, has become a useful and convenient surrogate endpoint of insulin resistance. Numerous studies have shown that the TyG index is closely associated with the hyperinsulinemic-euglycemic clamp and is indicative of metabolic illness, heart disease and NAFLD [6, 7]. Nonetheless, NAFLD insulin resistance also has close ties to obesity and visceral adiposity, not reflected by a single index like the TyG. To address this shortcoming, a new index known as the triglyceride-glucose-body mass index (TyG-BMI) has been introduced, in which BMI is i ncluded to reflect adiposity. It has been argued that TyG-BMI could be more metabolically risky and liver fat-accumulating than TyG itself since it incorporates both biochemical and anthropometric measurements [8, 9]. Research has produced more robust relationships between TyG-BMI and NAFLD prevalence, hepatic steatosis and fibrosis risk than with the TyG index alone, especially in Asian groups, where body fat is not distributed the same way as in Western groups [10]. Steatosis and fibrosis need to be assessed since the stage of fibrosis is the most relevant predictor of liver-related morbidity and mortality in NAFLD patients [11]. Although liver biopsy is regarded as the gold standard, it is an intrusive technique and cannot be used in mass screening. Non-invasive imaging methods like ultrasonography and elastography are becoming more important and predictors of risk based on biochemical measures remain cost-effective and useful for risk stratification, particularly in resource-constrained tertiary care units [12]. India is also experiencing a fast epidemiological change whereby there has been a growing prevalence of metabolic syndrome, obesity and type 2 diabetes, which are all causative of the NAFLD burden [13]. Therefore, it is of interest to compare the diagnostic strength of the TyG index and TyG-BMI index in NAFLD patients and to determine their association with the severity of hepatic steatosis and fibrosis.

## Materials and Methodology:

## Study design and setting:

The current study is a cross-sectional, retrospective study that collected information in the Department of Medicine at Government Medical College Jammu, a tertiary care teaching hospital in North India. The analysis involved reviewing patient records over one year.

## Population and sample size of the study:

Those who were included in the study were 500 patients who were diagnosed with non-alcoholic fatty liver disease (NAFLD) during the study period. The NAFLD diagnosis was performed using the imaging evidence of liver steatosis without a considerable alcohol intake and other established causes of chronic liver disease.

## Inclusion criteria:

[1] Adults aged ≥18 years

[2] Patients whose ultrasonography of the liver shows fatty liver.

[3] Availability of full biochemical and anthropometric information.

[4] Patients who were tested by some form of fibrosis evaluation by elastography or any form of valid fibrosis scoring.

## Exclusion criteria:

[1] Major alcohol intake (>20 g/day in female, >30 g/day in male) history.

[2] Viral hepatitis (HBsAg or anti-HCV positive)

[3] Autoimmune liver disease, drug-induced liver disease, or metabolic liver disease.

[4] Pregnant women

[5] Incomplete records or laboratory parameters missing in the patients.

## Data collection:

Medical records from hospitals and electronic databases were retrieved to obtain patient data. The next information was gathered:

[1] Demographic details (age, sex)

[2] Anthropometric (height, weight, BMI) measurements.

Laboratory parameters such as fasting blood glucose and fasting triglyceride levels.

[1] Liver function tests

[2] Hepatic steatosis ultrasonographic grading.

[3] When available, liver stiffness measurements of elastography.

## Calculation of indices:

Standard correct formulae were used to calculate the indices:

[1] TyG Index = ln (fasting triglycerides (mg/dL) times fasting glucose (mg/dL)/2).

[2] TyG-BMI Index = Body Mass Index (kg/m^2^) x TyG index.

## Assessment of steatosis:

The hepatic steatosis was graded on ultrasonography as:

[1] Grade I (mild)

[2] Grade II (moderate)

[3] Grade III (severe)

## Assessment of fibrosis:

Assessment of fibrosis was done based on values of liver elastography or validated fibrosis scoring systems and defined as:

[1] No/Mild fibrosis

[2] Significant fibrosis

[3] Advanced fibrosis

## Outcome measures:

The primary objectives were:

[1] To compare the change in TyG and TyG-BMI in patients with NAFLD.

[2] To assess their relationship with the extent of hepatic steatosis.

[3] To determine whether they are correlated with liver fibrosis

## Statistical analysis:

The information was entered into Microsoft Excel and processed using statistical software. Continuous variables were reported as means with standard deviation and categorical variables as percentages. Comparison between groups: because there were continuous variables, a Student t-test was used; for categorical variables, a chi-square test was used. Pearson or Spearman correlation coefficients were used to assess the association between the indices and the severity of steatosis/fibrosis. ROC curves were plotted to compare the diagnostic's performance. A p-value below 0.05 was found to be statistically significant.

## Results:

A total of 500 patients with NAFLD were included in the study conducted at Government Medical College, Jammu. Demographic, biochemical and imaging parameters were analyzed to evaluate the associations of TyG and TyG-BMI indices with hepatic steatosis and fibrosis. The cohort was predominantly male (57.2%). Overweight/obesity was common, with a mean BMI of 28.6 kg/m^2^. Nearly half of the patients had metabolic comorbidities-42.4% diabetes and 47.6% hypertension-indicating strong metabolic risk clustering typical of NAFLD populations. These baseline factors were significantly associated with higher TyG-BMI values (p = 0.01) (Table 1 - see PDF). Moderate steatosis was the most common finding (40.8%), while 21.6% had severe fatty infiltration. Fibrosis assessment revealed that 44.8% of patients had clinically relevant fibrosis (significant or advanced). Increasing fibrosis severity was associated with metabolic indices in a statistically significant manner (p <0.001), supporting the link between insulin resistance and liver damage (Table 2 - see PDF). The table demonstrates a gradual increase in both the TyG index and TyG-BMI index with increasing severity of steatosis. Patients with moderate steatosis showed higher mean values compared to those with mild steatosis, and this difference was statistically significant (p<0.001). Similarly, individuals with severe steatosis had the highest TyG and TyG-BMI values, indicating a strong positive association with disease severity. These findings suggest that elevated TyG and TyG-BMI indices are significantly associated with worsening steatosis, and may serve as reliable indicators for assessing the severity of fatty liver disease (Table 3 - see PDF). The table shows a progressive increase in both the TyG index and TyG-BMI index with advancing stages of fibrosis. Patients with significant fibrosis had higher mean values compared to those with none/mild fibrosis, and this increase was statistically significant (p<0.001). Similarly, individuals with advanced fibrosis demonstrated the highest TyG and TyG-BMI values, indicating a strong association with disease severity. Overall, the findings suggest that higher TyG and TyG-BMI indices are significantly correlated with increasing fibrosis severity, and may serve as useful non-invasive markers for fibrosis progression (Table 4 - see PDF).

## Discussion:

NAFLD is the hepatic expression of the metabolic syndrome and is closely interrelated with insulin resistance, dyslipidemia and obesity. The current retrospective cross-sectional study at Government Medical College Jammu has shown that the TyG and TyG-BMI indices increased most significantly as steatosis and fibrosis worsened, with the TyG-BMI index discriminating better. All these findings support the idea that adiposity-adjusted composite metabolic indices are more effective measures of hepatic disease severity. There is a growing consensus that the TyG index is a valid surrogate for insulin resistance and metabolic dysfunction. In the current study, TyG values increased with increasing grades of steatosis and fibrosis. The same was also observed in the cross-sectional study, where the prevalence of NAFLD increased significantly across TyG quartiles, with the lowest quartile (30.9%), the middle two quartiles (41.7-77.9%) and the highest quartile (86.4%). The rate of fibrosis also rose gradually, from the 13.5 to 26.1 percentile quartiles [13]. These results confirm our observation that hepatic deterioration is associated with metabolic deterioration. A different cross-sectional analysis indicated that higher TyG index values were independently associated with lower fibrosis scores, with adjusted odds ratios of 3.44 (in the highest quartile relative to the lowest) [14]. Likewise, the recent population-based studies of the United States provided evidence that the high level of TyG indices is positively correlated with the risk of liver fibrosis, especially in obese and metabolically impaired patients. Therefore, the current results are consistent with evidence from the worldwide literature that TyG reflects the burden of steatosis and fibrotic risk in the liver. Although TyG alone is a test of insulin resistance, the TyG-BMI index incorporates obesity into NAFLD pathogenesis, a core condition. TyG-BMI showed greater percentage differences between steatosis and fibrosis types in our study than TyG alone. This aligns with metabolic theory, which posits that visceral adiposity facilitates hepatic lipid deposition by exaggerating free fatty acid flux, inflammatory cytokine production and oxidative stress. Other clinical studies have also shown that composite metabolic indices are better than individual biochemical indices for identifying metabolically unhealthy individuals and those at risk of fatty liver disease [15]. The positive association of TyG-BMI with fibrosis (in our cohort) indicates that insulin resistance associated with adiposity might be the leading cause of fibrogenesis, rather than insulin resistance per se. This has clinical significance, as fibrosis, not steatosis, is the determinant of morbidity and mortality in NAFLD in the long run. In our study, almost 50 percent of patients had moderate to severe fibrosis, which shows that the disease burden is high in tertiary care. The correlation of metabolic indices with fibrosis in the present case confirms that metabolic inflammation triggers hepatic stellate cell and extracellular matrix deposition. The findings of other histopathological studies have also shown correlations between TyG-derived insulin resistance markers and the stage of fibrosis, suggesting that these indices can be used to identify patients at risk of progressing to cirrhosis and hepatocellular carcinoma [16, 17]. In another study, Zhu *et al.* reported that both TyG and TyG-BMI independently predicted NAFLD risk; however, TyG-BMI demonstrated superior diagnostic accuracy with higher AUC and favourable decision curve analysis in both cohorts. A TyG-BMI cut-off of <212.886 effectively ruled out NAFLD (sensitivity 80.6%, NPV 77.8%), while ≥251.741 ruled it in (specificity 90.7%, PPV 74.8%), suggesting TyG-BMI as a more clinically actionable screening tool, particularly where imaging is unavailable [18]. Since liver biopsy is invasive and imaging fails to detect early fibrosis, inexpensive screening tests such as TyG-BMI may be useful, especially in resource-constrained settings. The study benefits from a large sample size of 500 NAFLD patients, providing robust statistical power for comparative analysis. Conducted in a tertiary-care setting at GMC Jammu, it reflects real-world clinical data, with a direct comparison of TyG versus TyG-BMI indices against ultrasonographic and elastographic outcomes. Comprehensive fibrosis assessment further strengthens its diagnostic relevance for metabolic risk stratification. The retrospective design limits causal inference and introduces potential selection bias inherent to tertiary-care populations. The absence of histological confirmation represents a key methodological gap, while the single-center design limits generalizability to diverse populations and settings. Regardless of these shortcomings, the study contributes to the body of evidence in the region supporting the use of TyG-based indices to predict NAFLD severity.

## Conclusion:

Both TyG and TyG-BMI indices strongly correlated with hepatic steatosis and fibrosis severity in 500 NAFLD patients, with TyG-BMI demonstrating superior discriminatory capacity due to adiposity integration. TyG-BMI emerges as a simple, cost-effective, non-invasive screening tool for identifying high-risk NAFLD progression, particularly valuable in resource-limited settings. Prospective multicenter validation is recommended to establish clinical cutoffs and confirm its utility for fibrosis risk stratification in diverse populations.
